# Cancer Stem Cells in Pheochromocytoma and Paraganglioma

**DOI:** 10.3389/fendo.2020.00079

**Published:** 2020-02-25

**Authors:** Laura D. Scriba, Stefan R. Bornstein, Alice Santambrogio, Gregor Mueller, Angela Huebner, Julia Hauer, Andreas Schedl, Ben Wielockx, Graeme Eisenhofer, Cynthia L. Andoniadou, Charlotte Steenblock

**Affiliations:** ^1^Department of Internal Medicine III, University Hospital Carl Gustav Carus, Technische Universität Dresden, Dresden, Germany; ^2^Diabetes and Nutritional Sciences Division, King's College London, London, United Kingdom; ^3^Centre for Craniofacial and Regenerative Biology, King's College London, London, United Kingdom; ^4^Children's Hospital, University Hospital Carl Gustav Carus, Technische Universität Dresden, Dresden, Germany; ^5^Department of Pediatrics, Pediatric Hematology and Oncology, University Hospital Carl Gustav Carus, Technische Universität Dresden, Dresden, Germany; ^6^Université Côte d'Azur, INSERM, CNRS, iBV, Nice, France; ^7^Institute of Clinical Chemistry, University Hospital Carl Gustav Carus, Technische Universität Dresden, Dresden, Germany

**Keywords:** cancer stem cells, adrenal, pheochromocytoma, paraganglioma, hypoxia

## Abstract

Pheochromocytoma (PCC) and paraganglioma (PGL) are rare neuroendocrine tumors associated with high cardiovascular morbidity and variable risk of malignancy. The current therapy of choice is surgical resection. Nevertheless, PCCs/PGLs are associated with a lifelong risk of tumor persistence or recurrence. A high rate of germline or somatic mutations in numerous genes has been found in these tumors. For some, the tumorigenic processes are initiated during embryogenesis. Such tumors carry gene mutations leading to pseudohypoxic phenotypes and show more immature characteristics than other chromaffin cell tumors; they are also often multifocal or metastatic and occur at an early age, often during childhood. Cancer stem cells (CSCs) are cells with an inherent ability of self-renewal, de-differentiation, and capacity to initiate and maintain malignant tumor growth. Targeting CSCs to inhibit cancer progression has become an attractive anti-cancer therapeutic strategy. Despite progress for this strategy for solid tumors such as neuroblastoma, brain, breast, and colon cancers, no substantial advance has been made employing similar strategies in PCCs/PGLs. In the current review, we discuss findings related to the identification of normal chromaffin stem cells and CSCs, pathways involved in regulating the development of CSCs, and the importance of the stem cell niche in development and maintenance of CSCs in PCCs/PGLs. Additionally, we examine the development and feasibility of novel CSC-targeted therapeutic strategies aimed at eradicating especially recurrent and metastatic tumors.

## Introduction

The adrenal gland is composed of two main tissue types that establish a bidirectional connection; the catecholamine-producing chromaffin cells in the medulla and the primarily steroid-producing cells in the cortex. The inner medulla is derived from neuro-ectodermal cells of neural crest origin while the outer cortex is derived from the intermediate mesoderm. Close interactions between the two components are necessary for differentiation and morphogenesis of the gland (1, 2). Although highly heterogeneous, pheochromocytomas (PCCs) and paragangliomas (PGLs) are generally slow growing neural crest-derived tumors comprised of chromaffin cells arising at intra- and extra-adrenal locations, respectively (3). The tumors show a very high rate of germline or somatic mutations in a large number of genes (4, 5). When occurring at a young age, the tumors are often multifocal, suggesting development during embryogenesis from a common progenitor during neural crest migration to different sites of tumorigenesis (6).

## Stem Cells in the Normal Adrenal Medulla

The neural crest represents a migratory population of multipotent cells transiently present during embryogenesis, which gives rise to a diverse variety of cell types and tissues (7–9). At 11.5 days post coitum (dpc) in the mouse, early neural crest cells migrate near the dorsal aorta, and form the suprarenal ganglion. These progenitors will give rise to both chromaffin cells and neurons, which innervate the adrenal medulla. From 11.5 to 15.5 dpc, late neural crest cells migrate toward the dorsal root ganglion, where they acquire a Schwann cell precursor (SCP) fate and express the transcription factor SOX10, which is reported to be expressed in human, bovine and murine chromaffin progenitors and stem cells (10–12). SOX10-positive SCPs migrate along the nerves toward the medulla primordium, and give rise to over 50% of the adrenomedullary chromaffin cells (13). The majority of chromaffin cells of the Zuckerkandl organ, which is the largest extra-adrenal chromaffin body in mammals, are also generated from SCPs (14). Until recently, it was believed that sympathoblasts and chromaffin cells originate from a common progenitor (15), but the recent studies of SCPs show an earlier segregation of the two lineages (14). SCPs have lately attracted considerable attention due to numerous reports highlighting their role as multipotent progenitors generating various fates in the body (16–20). In addition to giving rise to chromaffin and mature Schwann cells, SCPs propagate to mesenchymal stem cells (21–23), endoneural fibroblasts (24), and melanocytes (25), which use the peripheral nerves to reach their specific locations in the developing embryo (18).

The postnatal adrenal medulla is composed of two main differentiated cell types: catecholamine-producing chromaffin cells, which represent the functional unit of the gland, and neurons stimulating the catecholamine production. A third cell type of the adrenal medulla has been identified as “sustentacular” or “support” cells. These cells are of glial nature and express S100B, GFAP, and Vimentin (26). Sustentacular cells are found in proximity to both chromaffin cells and nerve terminations, but their function has not been clarified yet. Sustentacular cell markers are expressed by Nestin-positive cells, proposed to be stress-responsive progenitors of the adrenal medulla (11). The cytoskeletal type VI intermediate filament Nestin was initially identified as a marker of neural stem and progenitor cells (27, 28), but was later shown to be expressed in a variety of tissues and other stem or progenitor cells (29). Nestin appears to be linked to essential stem cell functions including self-renewal/proliferation, differentiation and migration (29), and is expressed in progenitors of both the adrenal cortex and medulla (30, 31). Furthermore, Nestin seems to correlate with malignancy in several different cancer types (32). In the adrenal medulla, Nestin-positive cells overlap with CD133-positive cells and can differentiate into chromaffin cells and cells of the neural lineages (11). CD133 is expressed by progenitor/stem cells in several organs including the brain under healthy or cancerous conditions [for review see (33)], and expression of CD133 has also been demonstrated in primary cultures of human and bovine adrenal medullary cells grown under stem cell promoting conditions (10, 12).

## Tumorigenesis of PCCs and PGLs

Around 30–40% of PCCs/PGLs are hereditary due to mutations in close to 20 currently identified PCC/PGL susceptibility genes, whereas another 40–50% show somatic mutations in the same as well as other genes (34, 35). At the time of PCC/PGL diagnosis, the mean age of patients is 40–50 years, though 10–20% of all cases are diagnosed in children (36). PCCs/PGLs can be divided into two main clusters depending on their underlying mutations in any of the susceptibility genes: the pseudohypoxia-associated cluster 1 and the kinase signaling-associated cluster 2 (4, 34). A third WNT signaling-associated cluster due to translocation of *MAML3* has also recently been described (35), but remains poorly characterized. Genes most commonly contributing to cluster 1 PCCs/PGLs are those encoding the four subunits of the succinate dehydrogenase (SDH) enzyme, namely *SDHA, SDHB, SDHC*, and *SDHD*, and the SDH assembly co-factor *SDHAF2*. Other genes associated with a pseudohypoxic signature include *VHL, EPAS1, FH, MDH2*, and *EGLN1/2*. Chromaffin cell tumors arising due to mutations affecting SDH, but particularly SDHB, are predominantly aggressive and often malignant. These tumors and those due to *VHL* mutations often occur in childhood suggesting development during embryogenesis from a common stem cell/progenitor. According to the classical “two-hit” model, two mutations are a prerequisite for tumorigenesis resulting from loss of function mutations. In addition to the original germline/somatic mutation, tumorigenesis requires a second somatic mutation of the same gene (37). However, compared to other tumors PCCs/PGLs exhibit a low somatic mutation rate (35) suggesting that at least in pediatric tumors a single mutation is sufficient for tumorigenesis. Cluster 2 tumors include mutations in the *RET, NF1, TMEM127*, and *MAX* genes and are characterized by activated PI3K/AKT/mTOR and RAS/RAF/ERK downstream kinase and protein translation signaling pathways (38). These tumors almost always originate in the adrenals, and clinically they do not display a particularly aggressive behavior. Furthermore, they have more mature catecholamine secretory pathways and phenotypic features, and they tend to develop later in life than tumors due to cluster 1 mutations (6, 39).

Normal stem cells are regulated by extrinsic cytokines as well as by intrinsic genetic programs within their niche (40). This niche must be pliable to coordinate both homeostasis and repair; however, such flexibility can be distorted by chronic diseases and cancer. During embryonic development, especially before vascularization, cells exist in a relatively oxygen-poor environment. Consequently, oxygen sensing pathways play crucial roles in ensuring appropriate embryonic morphological development and survival (41). Similarly, intratumoral hypoxia provides a microenvironment that shields CSCs and stimulates their proliferation (42). Under changing oxygen levels hypoxia-inducible transcription factors (HIFs) activate genes that promote tolerance of hypoxia by decreasing the cellular requirements for oxygen and by increasing the supply of oxygen (43–45). This is potentially mediated by two HIF isoforms, HIF1α and HIF2α differentially coordinating migration, survival and differentiation of neural crest cells (46, 47).

The common denominator for the pseudohypoxic phenotype of all cluster 1 tumors involves HIF stabilization. It appears that stabilization of HIF2α rather than HIF1α is responsible for tumor development and the distinct phenotypic features of cluster 1 chromaffin cell tumors (47). Stabilization of HIF2α also provides the unifying mechanism responsible for the pseudohypoxic phenotypes of all cluster 1 PCCs/PGLs (48). Mutations in the *EPAS1* gene encoding HIF2α are almost always somatic, but still often involve a syndromic presentation including polycythemia (elevated volume of red blood cells in the blood) and somatostatinomas (49, 50). Although lacking the central pseudohypoxic footprint, the cluster 2 tumors relies on a glycolytic and glutaminolytic switch, necessary for cell proliferation and survival, as well as for chromatin remodeling. This means that even though genetically there is a high heterogeneity in PCCs/PGLs, the molecular pathways defining the three clusters are interrelated and all participate in developmental processes (51).

**Figure 1:**
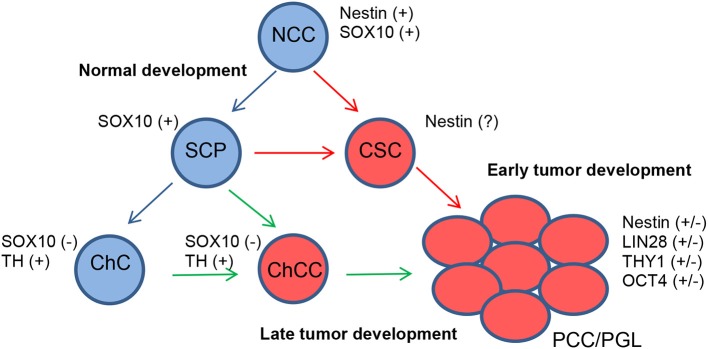
Proposed model for the development of PCCs/PGLs. Under normal conditions, neural crest cells (NCCs) differentiate into SCPs and finally chromaffin cells (ChCs) (blue arrows). In PCCs/PGLs, developing at an early age, somatic mutations in SCPs give rise to CSCs from where tumors develop (red arrows). Somatic mutations happening in ChCs also give rise to PCCs/PGLs via chromaffin cancer cells (ChCCs) but with a later appearance (green arrows).

Especially in cluster 1 tumors that develop early in life, mutated SCPs might be one of the initiating tumorigenic cell types since recent data on SCPs reveal that they can give rise to both adrenal and extra-adrenal chromaffin cells. Furthermore, PCCs and PGLs share diagnostic markers. Other tumor-initiating cell types could be chromaffin cells, sympathetic-like chromaffin cells or sympathoblasts (Figure 1).

Even if the chromaffin cells in the adrenal medulla and extra-adrenal tissues, like the Zuckerkandl organ, have the same origin, they are quite different at later developmental steps (14). This suggests that the earlier the mutation happens, the higher the risk for developing multifocal tumors. When the mutation arises later, the progenitor cell is already committed to giving rise to either intra- or extra-adrenal chromaffin tissue. This might also explain the differences in metastatic potential between PCCs and PGLs as about 10–20% of PCCs are metastatic, whereas up to 50% of PGLs are metastatic (52).

The high prevalence of multifocal tumors at a young age may indicate secondary somatic mutations before the settlement of migrating SCPs at different locations. In support of this concept, PGLs carrying somatic *EPAS1* (HIF2α) mutations are associated with mosaicism and identical mutations in multiple tumor sites, consistent with the timing of tumor development as a result of postzygotic mutations during early embryogenesis (53, 54). During embryonic development, a large proportion of sympatho-adrenal progenitors undergo programmed cell death. This is also in keeping with observations that paraganglial chromaffin tissue, and particularly the Zuckerkandl organ, are well developed in the late-stage human fetus and regress after birth with only vestigial clusters of cells persisting into adulthood (55). These extra-adrenal clusters of chromaffin cells may reflect the remnants of the hypoxia-sensing peripheral catecholamine system, which is more important during fetal development than in adulthood. This is different from the neural crest-derived carotid body that remains important throughout adulthood for oxygen sensing. In a physiological state, the carotid body responds to chronic hypoxia with hyperplasia and hypertrophy of its neural and vascular tissue components. In order to do this neural crest-derived progenitors in the carotid body retaining mesoectodermal differentiation potential are reactivated in a HIF-dependent manner (56–58).

## Cancer Stem Cell Markers Found in PCCs and PGLs

CSCs are self-renewing and due to their stem-like properties they may be more resistant to chemotherapy and radiation (59). As these cells are potentially at the basis of tumor formation and thereby a potential target for future therapies, a lot of attention has been focused on their existence and impact in several other cancers, especially in endocrine and neural tissues. Even though many of the PCC/PGL mutations are related to stem cell markers (51), there are few studies focusing on CSCs in PCCs/PGLs. Increasing evidence suggests that CSCs are reliant on low oxygen conditions, and therefore on HIF1α and HIF2α to maintain their stem cell features (47, 60). Recently, it was shown that only in CSCs the pluripotency-promoting transcription factors NANOG and SOX2 cooperate with MYC to regulate HIF2α, which leads to a decrease in P53 expression and reduces the levels of reactive oxygen species in CSCs thereby promoting stemness (61). This is supported by other studies showing that primarily HIF2α is activated in CSCs (62).

Often the results on the expression of stem cell markers in PCCs/PGLs are contradictory, likely reflecting tumor heterogeneity (Table 1). Though the molecular pathways in the different tumors might be related, the amount of the different cell types in the tumors and their levels of differentiation, remain variable, possibly due to their microenvironment (51). For example, the expression of OCT4 is inconsistent as a study by Looijenga et al. concluded that PCCs/PGLs are negative for OCT4 (63), yet a subsequent study by Alexander et al. showed strong and diffuse cytoplasmic staining of OCT4 in PCCs and metastases (64). Oudijk et al. analyzed the expression of relevant CSC markers in tissue microarrays of a large number of PCCs and PGLs and showed that frequently CSC marker expression was associated with cluster 1 *SDHx*-mutated tumors (65), though expression of CSC markers has also been observed in PGLs from patients without *SDHx* mutations (66). Contrary to the findings within other adrenal tumors, OCT4, CD133, and NANOG expression was not detected in any of the samples from Oudjik et al., however expression of other stem cell markers such as DLK1/PREF1, NGFR, LIN28, SOX2, and THY1 was observed in 12-40% of cases, whereas the expression of Nestin, SOX17 and CD117 was identified less frequently in 2–3% of cases (65). In contrast, a case study of a PCC in pregnancy showed high expression of Nestin (67), which, as CD133, has also been detected in PGLs (66). As Nestin marks sustentacular cells, it raises questions regarding their role in PCCs/PGLs. To date, the precise role of this cell type in the normal adrenal medulla and in PCCs/PGLs has not been clarified. Several studies demonstrated differences in the number of sustentacular cells in primary and metastatic PCCs (70, 71), and a case of a distinctive neoplasm thought to have originated from S100-positive sustentacular cells has previously been reported (72). These observations support the notion that sustentacular cells may have a role in tumor formation or metastasis of PCCs/PGLs. However, these results also show the paucity of studies analyzing the expression of CSC markers in all clusters of PCCs/PGLs.

**Table 1:** CSC markers in PCCs/PGLs.

**Marker**	**Subcellular location**	**Function**	**Expression in PCCs/PGLs (percentage of positive tumors)**	**References**
OCT4	Nucleus and cytoplasm	Transcription factor Important for maintaining pluripotency, forms a complex with SOX2, regulates NANOG	+(0–100%)	(63–65)
NANOG	Nucleus	Transcription factor Important for maintaining pluripotency	–	(65)
SOX2	Nucleus	Transcription factor Expressed in the developing CNS Important for maintaining pluripotency, forms a complex with OCT4	+(12%)	(65)
SOX17	Nucleus	Transcription factor Regulates embryonic development	+(2%)	(65)
Nestin	Cytoplasm	Type VI intermediate filament Marker of neural stem/progenitor cells	+(3%)	(65–67)
CD90 (THY1)	Cell membrane	N-glycosylated, glycophosphatidylinositol anchored cell surface protein Marker of various stem cells	+(40%)	(65, 66)
CD117 (C-kit)	Cell membrane	Tyrosine-protein kinase acting as a receptor for c-kit ligand. Involved in growth and development of the cerebral cortex	+(3–14%)	(65, 66, 68, 69)
CD133 (Prominin-1)	Cell membrane	Cell surface glycoprotein Plays a role in cell differentiation, proliferation and apoptosis	±	(65, 66)
CD271 (NGFR)	Cell membrane	Nerve growth factor receptor Can mediate cell survival as well as cell death of neural cells	+(19%)	(65)
LIN28	Nucleus, endoplasmic reticulum, cytoplasm	RNA binding protein enhancing translation of IGF-2 Regulates self-renewal of stem cells	+(24%)	(65)
DLK1 (PREF1)	Cell membrane	Protein Delta Homolog 1, transmembrane protein belonging to the EGF-like homeotic protein family Plays a critical role in differentiation processes	+(19%)	(65, 66)

## CSC Targeted Therapies

When possible, surgery is always the therapy of choice for non-metastatic PCC/PGL. With metastatic disease, a primary tumor resection is recommended followed by radiotherapy or classical chemotherapy [reviewed in (73)]. In the case of progression or if classical therapies are not possible or tolerated by the patient, targeted therapies might be considered. As most PCCs/PGLs show a high expression of somatostatin receptor subtype 2, radiolabeled somatostatin analogs have been investigated as a treatment option (74). Other targeted therapies with for example different tyrosine receptor kinase inhibitors are under investigation and show promising results (73). As PCCs/PGLs occasionally express CD117 (Table 1), the tyrosine kinase inhibitor imatinib has for example been tested on *in vitro* cultures of PGL cell cultures, where it was shown to inhibit growth of both *SDHx*-unrelated and related tumor cells. Furthermore, it prevented xenograft formation in mice transplanted with patient-derived cells (66).

CSCs are known to be more resistant to conventional chemo- and radio-therapy compared to non-CSC populations. Pre-clinical and clinical trials in multiple tumor types have targeted CSCs via surface markers, inhibition of developmental stem cell pathways, or ablation of CSC niches [reviewed in (75)]. In neuroblastoma (NB) a common link between signaling cascades involved in tumorigenesis is hypoxia, and in hypoxic regions of NB, HIF1, and HIF2 have been shown to be expressed in cells with stem-like features (76). NB is the most common extracranial pediatric solid tumor, which often arises in infants and children up to 5 years (77). It originates from embryonic neural crest cells during early embryogenesis and mutated SCPs are likely also in these tumors the initiator cells (14). In contrast to PCCs/PGLs, the expression of stem cell markers in NB have been more studied (78). The existence of CSCs in NB has been associated with malignancy, resistance to chemotherapy and recurrence (79), as in chemo- and/or radiation-resistant NB cancer stem-like cells an increased expression of the stem cell markers CD133, SOX2, ALDH1, Nestin, OCT4, and NANOG was observed (80). Additionally, populations of cells positive for Nestin, CXCR4, and OCT4 were increased in late-stage NB (81). It has been suggested that differentiation of such NB CSCs may render them more sensitive to therapeutic intervention (78). One way to induce cancer differentiation in NB is to use retinoic acid (82), and actually combined retinoic acid treatment and proteasome inhibition demonstrated the ability to inhibit tumor-sphere formation and apoptosis in NB CSCs. Also the expression of Nestin, SOX2, and OCT4 was reduced with this treatment (83). Other combination therapies for NB CSCs showed promising results as well [reviewed in (78)]. Because of the similarity in origin and CSC marker expression between NB and PCCs/PGLs, these therapies might also be an option for the treatment of PCCs/PGLs.

In glioblastoma, the epidermal growth factor receptor (EGFR) antagonist nimotuzumab targeting CD133 has been shown to radiosensitize subpopulations of cells (84). Furthermore, anti-EGFR therapy in combination with conventional therapy has shown promising results in patients with epithelial cancers (85–87). Furthermore, in a cohort of advanced basal cell carcinoma patients, Von Hoff *et al*. demonstrated a favorable response to treatment with a small-molecule inhibitor of the hedgehog pathway (88). Other studies have investigated the NOTCH and WNT signaling pathways in multiple solid tumor patient populations (89). Stem cell specific niche constituents and their cognate receptors such as fibronectin and the fibronectin receptor in acute myeloid leukemia and breast cancer, respectively, have also been targeted and demonstrated strong anti-tumorigenic activity (90, 91). Since many of these targets, as e.g., NOTCH1 and CD133, are also expressed in metastatic PCCs/PGLs (66), the CSC-targeted therapies mentioned above might also be an opportunity for the treatment of these tumor types.

## Conclusion

While the existence of CSCs has been observed not only in PCCs/PGLs but also in several other endocrine and neural tumor types, the precise role of these cell types in tumor formation and homeostasis remains largely unknown. PCCs/PGLs include a subset of tumors in a pseudohypoxic state. The signaling and tissue microenvironments described in this state seem to encourage cell proliferation and survival and may even support persistence of cells expressing embryonic stem cell markers. Expression of several stem cell markers appears to be associated with PCC/PGL mutations, although the precise nature of this possible relationship needs to be further elucidated. Additionally, sustentacular cells may play a role in tumor development or invasiveness. However, there is still a need for thorough characterization of these cells in tumor formation. Finally, investigations into the homeostatic role of adult stem cells within the normal adrenal medulla in comparison to the tumor state may help understand the contribution of CSCs to PCCs/PGLs, which might lead to new therapies targeting CSCs in recurrent and metastatic PCCs/PGLs. Until now, CSC targeted therapies have not been employed for the treatment of PCCs/PGLs. However, due to similarity in the expression of CSC markers known from other tumor types, where CSC targeted therapies have shown promising results, these therapies might be an option for PCCs/PGLs as well. In conclusion, characterization of CSCs in PCCs/PGLs and the regimen of CSC targeted therapies of these tumors require further research.

## Author Contributions

LS and CS wrote the first draft of the manuscript. ASa, CA, GE, and CS wrote sections of the manuscript. LS, SB, ASa, ASc, GM, AH, JH, BW, GE, CA, and CS contributed to manuscript revision, read, and approved the submitted version.

### Conflict of Interest

The authors declare that the research was conducted in the absence of any commercial or financial relationships that could be construed as a potential conflict of interest.
